# Unusual Diversity of Sex Chromosomes in African Cichlid Fishes

**DOI:** 10.3390/genes9100480

**Published:** 2018-10-04

**Authors:** William J. Gammerdinger, Thomas D. Kocher

**Affiliations:** 1 Institute of Science and Technology (IST) Austria, Am Campus 1, 3400 Klosterneuburg, Austria; 2 Department of Biology, University of Maryland, College Park, MD 20742, USA; tdk@umd.edu

**Keywords:** sex chromosome evolution, African cichlids, sex chromosomes

## Abstract

African cichlids display a remarkable assortment of jaw morphologies, pigmentation patterns, and mating behaviors. In addition to this previously documented diversity, recent studies have documented a rich diversity of sex chromosomes within these fishes. Here we review the known sex-determination network within vertebrates, and the extraordinary number of sex chromosomes systems segregating in African cichlids. We also propose a model for understanding the unusual number of sex chromosome systems within this clade.

## 1. Introduction

Sex chromosomes control what is arguably the most important developmental decision, namely whether to develop as a male or a female. This decision has profound implications for the life history of the organism and, ultimately, its reproductive success. Sex chromosomes carry sex-determination genes that alter the earliest stages of gonadal differentiation. They may also carry a number of sexually antagonistic alleles [1,2] and a myriad of repetitive elements that further alter the fitness of individuals. It is therefore no surprise that sex chromosomes frequently play an important role in adaptation, through faster-X evolution [3], and in speciation, through Haldane’s rule [4,5].

Until recently, sex chromosomes were identified primarily by light microscopy, a technique that can only identify heteromorphic sex chromosomes. Most such differentiated sex chromosomes are old, and have diverged substantially from the autosomal pairs from which they are derived. Highly differentiated Y- or W-chromosomes have already lost many ancestral genes, and also gained a large number of repetitive sequences, relative to the ancestral autosome [6]. The earliest stages of sex chromosome evolution are less well studied. Only in recent years have advances in DNA sequencing allowed us to identify relatively undifferentiated, homomorphic sex chromosomes in the earliest stages of sex chromosome evolution in a wide variety of non-traditional model organisms [7]. 

### 1.1. The Canonical Model of Sex Chromosome Evolution

New sex chromosomes are thought to arise from the interplay between sexually antagonistic variation and mutations that alter the mechanisms of sex-determination [6,8,9]. A novel master sex-determination allele may emerge near a region of sexual antagonism, but will only rise to high frequency if it arises on a haplotype with a net favorable combination of sexually antagonistic alleles [10]. Mechanisms that reduce recombination, such as inversions, are then selectively favored because they maintain tight linkage between the sex-determination locus and nearby sexually antagonistic loci, as observed in cichlids and guppies [2,8,11]. Because these regions lack recombination, they also accumulate deleterious mutations following processes such as Muller’s ratchet [12]. Over time, recombination may also be reduced in adjacent regions, creating divergent evolutionary strata of varying ages between the newly evolved Y (W) and the X (Z) chromosome. Additional mutations gradually degrade the original gene content of the new Y (W) chromosome, leaving behind only a ghost of the original autosome [6,7,13].

### 1.2. Ancient Sex Chromosomes of Mammals and Birds

The evolutionary divergence of sex chromosomes has been most intensively studied in therian mammals. The XY system controlled by *Sry* arose ~181 million years ago (MYA) and has been retained in most mammalian lineages [14,15]. However, notable exceptions do exist. Some rodents, including the Japanese spinous country rat (*Tokudaia osimensis*) and the Transcaucasian mole vole (*Ellobius lutescens*), have lost the Y-chromosome and have only a single X-chromosome in both males and females [16,17,18]. Meanwhile, the closely related species *E. tancrei* and *E. talpinus* also lack a Y-chromosome, but have two X-chromosomes in both males and females [19,20,21].

The data also indicate an ancient origin of the ZW sex chromosome system found in avian lineages, dating back to ~137 MYA [15]. While the evidence is not yet conclusive, sex-determination in birds appears to be controlled by the presence of two copies of *DMRT1*, one on each of the Z-chromosomes in males [22,23]. Unlike mammals, there appears to be considerable variation in the magnitude of sex chromosome decay among bird lineages. Several ratite lineages have homomorphic sex chromosomes, while the sex chromosomes of carinates are usually heteromorphic [22,23,24,25]. Nonetheless, it seems that few, if any, transitions in the sex-determination mechanism have occurred during the radiation of birds.

However, there is reason to think that the stability of sex-determination mechanisms in mammals and birds is the exception rather than the rule. Molecular studies have revealed numerous sex chromosome transitions during the evolution of geckos [26]. Similarly, work on invertebrates has revealed an exceptional diversity of sex chromosome systems [27,28]. In this review, we focus on the recent explosion of knowledge about the sex-determination systems within teleost fishes.

## 2. Diversity of Sex Chromosomes in Fishes

In contrast to mammals and birds, cytogenetic studies have revealed unexpected diversity in the sex chromosome systems of fish. Only about 10% of the fishes that have been characterized have heteromorphic sex chromosomes [29]. The sex chromosome complements are diverse and include XY, ZW, XO, ZO, and complex XY systems [27]. Many species with homomorphic sex chromosomes are nevertheless male or female heterogametic, suggesting hidden diversity in the mechanisms of sex-determination. In a few families, including Cyprinidae [30,31], Poeciliidae [32], Loricariidae [33,34], and others [29], both male and female heterogametic species have been identified. While the data are still too sparse to definitively quantify the rates and patterns of transition between these sex chromosome systems, some trends are beginning to emerge, such as a bias toward XY systems over ZW systems and toward genetic sex-determination over environmental sex-determination [35].

### 2.1. Molecular Basis of Sex-Determination in Ricefish

**Figure 1:**
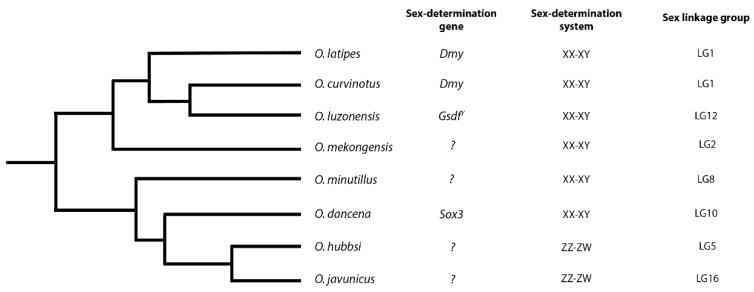
Phylogeny of *Oryzias* and their sex chromosome systems. Tree adapted from Takehana et al., 2005; Setiamarga et al., 2009; and Myosho et al., 2012 [44,45,47].

The karyotypes of ricefish (genus *Oryzias*) are homomorphic [36,37], but molecular studies have revealed a rich assortment of heterogametic sex chromosome systems (Figure 1). The sex-determination gene in medaka (*Oryzias latipes)* was genetically mapped to linkage group 1, where a male-specific duplication of *Dmrt1* was found [38,39]. Subsequent studies of other *Oryzias* species have revealed additional master sex-determination loci, including a Y-specific copy of *Gsdf* on linkage group 12 and a change in a *cis*-regulatory sequence of *Sox3* on linkage group 10 [40,41,42,43]. In other species from this genus, sex-determination has been mapped to linkage groups 2, 5, 8, and 16. This extraordinary diversity of sex-determination genes within *Oryzias* has arisen in only 30–60 million years [44,45,46]. 

These results from *Orzyias* allow us to make several general points. First, karyotypic surveys have undoubtedly missed most of the diversity of sex-determination mechanisms in fish species. The sex-determination gene, and the linkage group on which it resides, can be different among species with homomorphic sex chromosomes. Second, replacement of the sex-determination gene does not always result in a change from a male heterogametic system to a female heterogametic system, or vice versa. Most of the transitions in *Oryzias* are from one XY system to another XY system on a different linkage group. Third, these studies have identified new sex-determination genes that were not previously associated with the gene regulatory network for vertebrate sex-determination. This suggests either that the vertebrate gene network includes a larger number of genes, and/or that the set of genes participating in the network changes as the structure of the regulatory interactions evolves. 

Positional candidates for the sex-determination gene have been identified in several additional fish species. A duplication of *anti-Müllerian hormone* appears to be responsible for sex-determination in the Patagonian pejerry (*Odontesthes hatcheri*) [48]. A Y-specific missense mutation in *anti-Müllerian hormone receptor 2* has been identified in the fugu (*Takifugu rubripes*) [49]. Disruptions to the coding sequence of *growth differentiation factor 6* are found on the Y-chromosome of turquoise killifish (*Nothobranchius furzeri*) [50]. The sex of rainbow trout (*Oncorhynchus mykiss*) is determined by *sdY*, a divergent gene duplicate of *interferon regulatory factor 9* [51]. This locus is apparently responsible for sex-determination in many other salmonid species, but remarkably has translocated to several different linkage groups during speciation [52]. These genes likely represent just a fraction of the nodes in the teleost sex-determination gene regulatory network that can mutate to become top-level sex-determination genes.

### 2.2. Cichlid Sex Chromosomes

**Figure 2:**
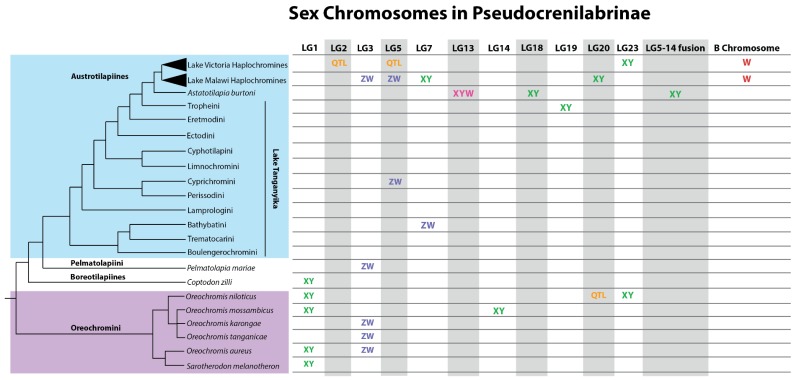
Overview of sex chromosome systems among African cichlids. Chromosome assignments based upon *O. niloticus* linkage groups (LG). Tree adapted from Meyer et al. (2015), Dunz and Schliewen (2013), Klett and Meyer (2002), and Nagl et al. (2001) [53,54,55,56]. QTL: Quantitative Trait Locus.

It is against this background that we now describe the extraordinary diversity of sex chromosomes that have been discovered among African cichlid fishes. Within the past decade there has been a surge in publications documenting the extraordinary variety of sex chromosomes among African cichlid fishes. To date, there have been more than a dozen different systems isolated on more than ten different chromosomes from this clade (Figure 2). Here, we briefly review each sex chromosome system, and then discuss a model that might explain the rich diversity of sex chromosomes within cichlid fishes.

The family Cichlidae emerged during the radiation of Perciformes ~100 MYA. The phylogeography of the clade strongly suggests a radiation prior to the breakup of Gondwana [57,58,59], but some molecular clock analyses suggest a more recent divergence [60,61]. The latest analyses suggest that the African cichlid clade Pseudocrenilabrinae emerged approximately 45 MYA [62]. 

The sex chromosomes of the deepest cichlid lineages have yet to be characterized. Here we focus on the Haplotilapiines [54,63], which include the astonishing radiations of species in the rivers and lakes of East Africa. The radiation of these species began 40 MYA. We begin with the “tilapia”, a paraphyletic group which includes the Oreochromini, Pelmatolapini, and the Boreotilapiines [54].

#### 2.2.1. Oreochromini

The first cichlid sex-determination system to be mapped, using bulked segregant analysis of microsatellites, was an XY locus on linkage group (LG) 1 of the Nile tilapia, *Oreochromis niloticus* [64]. Additional mapping has been performed with amplified fragment length polymorphism (AFLP) markers [65,66], microsatellites [67], and restriction site-associated DNA sequencing (RAD-seq) [68,69] to confirm this system. Whole-genome resequencing of male and female DNA pools identified a putative inversion encompassing ~8.8 Mb around the sex-determination locus [70]. The sex-associated region contains at least 164 missense alterations in protein-coding genes and these missense mutations are spread uniformly across the sex-associated region [70,71]. Early karyotype work had led to speculation that the largest chromosome (corresponding to LG3) was the sex chromosome in *O. niloticus* due to incomplete pairing during meiosis, a characteristic of nonhomologous sequences often associated with sex chromosomes [72,73,74,75]. However, more recent work has demonstrated that the mapped sex chromosome system on LG1 does not correspond to this large chromosome [66]; thus, it seems likely that this incomplete pairing was the result of highly repetitive regions residing on LG3 [76] and not a lack of homology between sex chromosomes. A recent RAD-seq experiment investigated the appearance of LG1 XX males in several families of *O. niloticus* that had shown skewed sex ratios (64% to 93% male) [69]. This experiment identified a quantitative trait locus (QTL) on LG20 that biased the sex ratio of LG1 XX individuals toward male [69]. 

A very similar LG1 XY system is found in *Sarotherodon melanotheron* [71]. The sex-associated region of LG1 includes 99 missense mutations in protein-coding genes. An additional region on LG1 and a second sex-linked region that corresponds to LG22 of the *O. niloticus* reference genome show slightly less differentiation, suggesting the presence of evolutionary strata of different ages [71]. However, within each of these regions there appears to be a uniform distribution of differentiation. Despite the significant sequence divergence and the apparent translocation from LG22 to LG1, the karyotypes of *O. niloticus* and *S. melanotheron* show no evidence of heteromorphic sex chromosomes [77,78]. 

The blue tilapia (*Oreochromis aureus*) segregates a ZW system on LG3 [67,79,80]. Interestingly, some stocks simultaneously segregate the ZW system on LG3 along with the previously described XY system on LG1. When both loci are segregating in a family, the LG3 W-chromosome is epistatically dominant to the Y-chromosome on LG1 [80]. Whole-genome scans from pools of males and females from *O. aureus* have demonstrated a high level of differentiation across the LG3 sex chromosome pair [76] despite being karyotypically homomorphic [78,81]. An additional karyotype from this species appeared to show evidence of incomplete pairing on two pairs of chromosomes, one of which was LG3 [82]. However, this incomplete pairing on LG3 appeared similar to the previously discussed incomplete pairing on LG3 in *O. niloticus* and it is thus unclear if this is the result of divergence in single-copy sequences or repetitive elements. The second incomplete pairing chromosome could represent the LG1 XY system since it also segregates in this species, but this possibility has not been explored further [82]. Association mapping with microsatellites identified similar LG3 ZW systems in *Oreochromis karongae* [67] and *Oreochromis tanganicae* [83]. Karyotypes from *O. karongae* also confirmed a lack of heteromorphic sex chromosomes [84,85].

A second XY system, on LG23, has been detected in several species of *Oreochromis*. Early work on this system noted a microsatellite, UNH216, on LG23 that was shown to correlate with a sex ratio distortion [86] in a meiogynogenetic line of *O. aureus*. QTL studies from the F_2_ of an interspecific cross between *O. aureus* and *O. mossambicus* also found associations of sex with markers on LG1, LG3, and LG23 [79,87]. More recently, some strains of *O. niloticus* have been described as segregating an XY system from LG23 that was originally mapped using microsatellite markers [88,89]. Subsequent work showed that the Y-chromosome has a duplication of the *anti-Müllerian hormone* gene [90,91]. One of the Y copies has an insertion that results in a premature stop codon, while the other Y copy has a missense single nucleotide polymorphism (SNP). A knock-out of the gene copy with the missense SNP was sufficient to cause male-to-female sex reversal [91]. To date, this is the only cichlid sex-determination gene that has been characterized at a molecular level. The epistatic interactions between this locus and the LG1 XY and LG3 ZW system are currently unknown.

Finally, a recent genome scan from *O. mossambicus* revealed a high density of XY-patterned SNPs uniformly spread across the first 10 Mb of LG14 [92]. This likely represents a young sex chromosome system, since there are only 69 mutations within this region that create missense mutations in their corresponding proteins. A previous microsatellite survey of this species revealed the influence of the LG1 XY and LG3 ZW systems, but did not test for markers on LG14 [67]. Karyotype analysis did not reveal any heteromorphic sex chromosomes in *O. mossambicus* [81].

#### 2.2.2. Boreotilapiines

The Boreotilapiines are widespread across rivers and lakes of West Africa and include the tribes Coelotilapiini, Heterotilapiini, Coptodonini, and Gobiocichlini [54,93]. Genotyping of microsatellites in a family of *Coptodon zillii* identified an XY sex-determination locus on LG1 [67]. This locus might be homologous to the LG1 system found in Oreochromini. Similar to *S. melanotheron*, this system also showed elevated levels of differentiation on LG22, suggesting a recent translocation from LG1 to LG22 in the *O. niloticus* lineage that was used to generate the reference genome [92]. Whole-genome resequencing of males and females from this species revealed more than 250 missense mutations uniformly spread across this sex chromosome pair, some of which are predicted to have deleterious impacts on their associated proteins [92]. Karyotype studies of *Coptodon zillii* [94,95], *C. rendalli* [95,96,97], *C. congica*, and *C. guineensis* [98] have not revealed any heteromorphic chromosome pairs in this clade.

#### 2.2.3. Pelmatolapini

The tribe Pelmatolapini ranges from West Africa to as far south as Angola. The placement of this tribe into the Boreotilapini or Austrotilapiniini has been unclear due to a hypothesized ancient hybridization event [54,63]. Karyotype evidence from this tribe is limited to *Pelmatolapia mariae*, but no study has revealed heteromorphic sex chromosomes [81,95,98]. Genotyping of microsatellites revealed a ZW system on LG3 in *Pelmatolapia mariae* [67]. Whole-genome resequencing of males and females confirmed the presence of a sex chromosome on LG3, but was unable to determine if the system was a ZW or XY system [92]. 

It is unclear whether the LG3 ZW sex chromosome systems in the Pelmatolapini and Oreochromini represent a shared ancestral polymorphism or if they represent convergent evolution. Studies indicate that LG3, the largest chromosome in these species, has a high density of repetitive elements [76,99]. Repetitive element density has been reported to be negatively correlated with recombination in many lineages [100,101,102]. A low rate of recombination might facilitate the repeated invasion of novel sex-determination alleles, because novel sex-determination alleles might be more tightly linked to sexually antagonistic loci that would help drive them to fixation. 

#### 2.2.4. Austrotilapiines 

Lake Tanganyika is the oldest of the African Great Lakes, dating back to 9–12 MYA [103]. It contains radiations of 12–16 major lineages of Austrotilapiines (sensu Schwarzer et al., 2009 [63]). These tribes encompass more than 250 species [104] which have radiated over the last 5–10 MY [60,105]. Despite this diversity, there has been relatively little work published on the sex chromosome systems of these lineages. We recently sampled species from three cichlid tribes in Lake Tanganyika and discovered a new sex chromosome in each tribe that we examined [106]. 

The tribe Bathybatini is one of the oldest lineages in Lake Tanganyika [107,108,109]. Using a genome resequencing approach, we found a ZW system on LG7 in *Hemibates stenosoma* [106]. The sex-linked region encompasses 37.5 Mb, with more than 1900 ZW-patterned missense mutations in their associated proteins spread uniformly across the sex-associated region.

The tribe Cyprichromini arose as part of the primary lacustrine radiation in Lake Tanganyika [107]. We used whole-genome resequencing to uncover a highly differentiated LG5 ZW system in *Cyprichromis leptosoma*. We also identified ZW differentiation on LG13, which likely indicates a translocation onto LG5, relative to the structure of the *O. niloticus* reference genome used for the analysis. It is unclear if this putative translocation occurred prior to or after the emergence of this sex-determination system since karyotypes are not yet available for this species to reveal whether it has homomorphic or heteromorphic sex chromosomes. However, the region corresponding to LG13 appears to have less differentiation and, thus, it appears to be a younger stratum. Together, these two regions had more than 1800 missense mutations in their associated proteins. The differentiation between males and females in this species extends across ~26 Mb of LG5 and ~1.1 Mb of LG13, and while the level of differentiation is different between the two regions, the differentiation within each region is uniform. Due to the high threshold used to determine the regions of highest differentiation, much of LG13 is not captured in this estimate since it shows lower levels of divergence, but the region of differentiation on LG13 may extend over as much as 10 Mb [106]. This system and the LG5 ZW system observed in Lake Malawi haplochromines (discussed below) are likely convergent [106].

A survey of the karyotypes of other tribes from Lake Tanganyika, including Lamprologini, Limnochromini, and Eretmodini, found no evidence of heteromorphic sex chromosomes [110].

#### 2.2.5. Haplochromines

The tribe Haplochromini also emerged during the primary lacustrine radiation in Lake Tanganyika [107] approximately 3–6.8 MYA [111,112]. They are now found throughout the lakes and rivers of East Africa [113,114]. This lineage has undergone additional notable radiations after entering Lake Malawi and Lake Victoria [113]. We discuss each of these radiations separately in the sections below.

##### Lake Tanganyika Haplochromines

The Tropheini are a monophyletic tribe nested within the Haplochromini that likely arose after a riverine lineage colonized Lake Tanganyika approximately 3–6.8 MYA [112,113]. While the sex chromosomes of this tribe have not been extensively investigated, a genome scan comparing males and females from *Tropheus* sp. “black” revealed extensive divergence on LG19 corresponding to an XY system. We found a region of decay totaling ~19 Mb in which there were >2000 XY SNPs uniformly spread across the sex-associated region causing missense mutations in their associated proteins [106]. Unfortunately, we are not aware of any karyotype data from this tribe.

*Astatotilapia burtoni* is found in the tributaries of Lake Tanganyika. Recent work has illuminated a diverse array of sex chromosome systems within this species. Two studies using a RAD-seq approach in *A. burtoni* found an XY system on a chromosomal fusion of LG5 and LG14 [115,116]. One of these studies also reported a weak, strain-specific XY signal from LG18 [115]. The other found an XYW system residing on LG13 [116]. A cytogenetic study did not report any sign of heteromorphic sex chromosomes in this species [81].

##### Lake Malawi Haplochromines

Lake Malawi cichlids have radiated into a flock of more than 700 species over the last ~0.5–2 MY [60,104,112,117,118,119]. Work on Lake Malawi haplochromines has so far identified at least five sex chromosome systems. 

Linkage mapping in the F_2_ offspring of a hybrid cross between *Labeotropheus fuelleborni* and *Metriaclima zebra* localized the sex-linked orange-blotch color phenotype to LG5 [120]. Subsequent genetic and association mapping confirmed the location of this ZW system, which is found in several genera of rock-dwelling cichlids in Lake Malawi [2,121,122]. 

Genotyping of microsatellites in lab-reared families of various *Metriaclima* species detected an XY system on LG7 [121]. This system was confirmed and further localized by additional mapping studies [123,124]. Several species in Lake Malawi segregate both the LG7 XY system and the LG5 ZW system and, in these species, the LG5 W allele is epistatically dominant to the LG7 Y [121]. An additional QTL study of a hybrid cross confirmed the LG5 ZW (23.50 percent variance explained (PVE)) and LG7 XY (10.83 PVE) systems [122]. This same study also detected a QTL of lesser effect on LG20 (8.14 PVE) and several other QTL that each explained less than 5% of the observed variance in sex-determination, including a ZW system on LG3 [122].

Lastly, a female-limited B-chromosome has been identified in species of *Metriaclima*, *Melanochromis*, and *Labeotropheus* [125]. B-chromosomes are supernumerary chromosomes that are not necessary for an organism’s survival and are found in some but not all individuals within a population. B-chromosomes are often replete with repetitive elements derived from the other chromosomes, which make them difficult to characterize [126,127]. The female-specific B-chromosome in Lake Malawi haplochromines was identified by scanning whole-genome resequencing runs for regions with unusually high copy number variants and confirming these regions with karyotype data [125]. Some, but not all, females in the population carry a single B-chromosome, which seems to function as a female-determining system that is epistatically dominant to the LG7 Y. 

##### Lake Victoria Haplochromines

Lake Victoria is the youngest of the Great Lakes. The radiation of 700 cichlid species that currently inhabit the lake dates back less than 300,000 years [112,119,128]. Relatively little work has been published identifying sex chromosomes from the Lake Victoria cichlids. Mapping in the F_2_ of an interspecific cross between *Haplochromis chilotes* and *Haplochromis sauvagei* identified QTL on LG2 and LG5 that explained 10% and 9.4% of the variance observed, respectively, but did not report if these loci were XY or ZW systems [129]. A recent study of *Pundamilia* also employed a QTL approach to map an XY sex-determination system to a 1.9 Mb region on LG23 which contains the *anti-Müllerian hormone* gene [130]. 

Finally, many cichlids in Lake Victoria also carry B-chromosomes [131]. In most species the B-chromosomes are found in both males and females, but in *Lithochromis rubripinnis* the B-chromosome was reported to be female-specific. Crosses in which neither the male or female carried a B-chromosome produced a ~50:50 sex ratio. Crosses involving females that carried one or more B-chromosomes produced 74–100% female offspring, suggesting that the B-chromosome has a feminizing effect in this species [131]. 

#### 2.2.6. Remarkable Diversity of Sex Chromosomes

This diverse array of sex chromosome systems in cichlids illustrates how rapid the transitions from one system to another can be. We have described more than a dozen sex chromosomes that have evolved during the recent evolution of East African cichlids. This is remarkably more than the number of currently described sex chromosomes that have evolved over a similar timescale in other vertebrate systems. Whether this means unusual mechanisms are at play in cichlids is not clear. Many vertebrate lineages have not been as extensively studied as cichlids. Also, the extraordinarily high species diversity of cichlids may allow us to detect more frequent transitions in sex-determination. The diversity of cichlid sex chromosomes creates an exceptional opportunity to unravel the patterns of sex chromosome divergence and the mechanisms responsible for sex chromosome transitions. 

#### 2.2.7. Stages in The Evolution of Sex Chromosomes

It is clear that many of these sex chromosome systems are still in the early stages of sex chromosome evolution. All of the cichlid sex chromosome systems that have been karyotyped are still visually homomorphic, but at the molecular level, we can classify the various systems according to the size of the chromosome region involved in sex-determination. For example, the LG7 XY system in Lake Malawi cichlids appears to involve a single gene, with no signs of inversion or divergence in adjacent regions. Likewise, the LG23 XY system in *O. niloticus* appears to be due to a small tandem duplication of a single gene and it thus seems likely that these species represent an early stage in sex chromosome evolution. Meanwhile, other sex chromosomes involve only a small part of the chromosome. For example, the LG1 XY locus in *O. niloticus* shows sequence differentiation across a region of only a few megabases. This likely represents a slightly older stage in the evolution of sex chromosomes.

Several of the sex chromosomes we have identified show high levels of differentiation across most of the chromosome. In *O. aureus*, divergence between the Z and W extends across most of LG3 [76]. The Z–W differentiation in *H. stenosoma* encompasses most of LG7 [106]. In *Tropheus,* the high X–Y differentiation extends over roughly two-thirds of LG19 [106]. There is no evidence for multiple evolutionary strata in any of these species, suggesting a singular event that reduced recombination on these sex chromosomes. 

Finally, three of the systems involve chromosome fusions/translocations. The Z–W differentiation on LG5 in *C. leptosoma* is uniform, consistent with a single evolutionary stratum. However, the Z–W differentiation on LG13 is noticeably less than on LG5, consistent with the idea that a portion of LG13 was translocated to LG5 relatively recently. Alternatively, the region that corresponds to LG13 in the *O. niloticus* reference genome may have translocated to LG5 deeper in the past of this lineage and this region has just recently been engulfed by an expansion of the region of reduced recombination and thus appears to be a younger stratum. The XY system in *S. melanotheron* derives mostly from LG1, but also includes a recent evolutionary stratum from LG22 [71]. The XY system in *A. burtoni* maps to a fusion of LG5 and LG14 and most of the differentiation is confined to LG5, but the density of sex-associated SNPs suggests the presence of multiple evolutionary strata on this chromosome [115]. 

It is not clear whether this molecular classification aligns well with the canonical model of sex chromosome evolution developed from studies of mammalian systems. So far, only a few cichlid sex chromosomes show evidence of multiple evolutionary strata. Even when large segments of the chromosome are involved, the magnitude of differentiation is usually similar across the entire sex-linked region. However, this could be due to their young age, as the evolutionary strata observed in mammals and birds often arose tens of millions of years apart [15].

## 3. Why Do Cichlids Have so Many Sex Chromosome Transitions?

The canonical model, developed primarily from studies of mammals, predicts that sex chromosomes will grow through the addition of evolutionary strata, leading to an evolutionarily stable and heteromorphic pair of sex chromosomes. Sex chromosome evolution in cichlids does not seem to follow this progression. Rather, cichlid sex chromosomes are homomorphic, and most lineages experience frequent sex chromosome replacement. It is not clear which pattern is more frequent in nature, but it still begs the question why the pattern is different among lineages.

The “hot-potato model” may be helpful in explaining frequent sex chromosome replacement [132]. This model envisions novel sex-determination alleles being driven to fixation by linked, sexually antagonistic alleles. However, as selection reduces recombination within these regions, they begin to accumulate deleterious mutations which reduce the fitness of the young sex chromosome [133]. At some point, any fitness advantage gained from the resolution of sexual conflict may be negated by the accumulation of deleterious alleles on the young sex chromosome. The system is then susceptible to invasion by a new sex chromosome system. The result could be an endless succession of ephemeral genetic mechanisms for sex-determination [132]. 

This process may explain the diversity and frequent turnover of sex chromosome systems in cichlids. It might also help to explain the polygenic sex-determination systems found in many cichlids. Although theory suggests that polygenic systems of sex-determination should not persist [134], the LG1 XY and LG3 ZW systems may have remained polymorphic for several million years in the Oreochromini. In Lake Malawi, some populations of *Metriaclima* are polymorphic for at least three sex chromosome systems (LG5 ZW, LG7 XY, and a B-chromosome). Persistence of these polymorphisms may depend a combination of drift and frequent changes in fitness due to the appearance of new sexually antagonistic or deleterious mutations on the sex-determination haplotypes.

How do we explain the existence of older, heteromorphic sex chromosomes like those of therian mammals and carinate birds? We suggest that the “hot-potato model” may best explain the earliest stages of sex chromosome evolution. During this time, the selective advantages offered by a new sex-determination locus with linked sexually antagonistic loci is relatively small and the genetic load from the accumulation of deleterious mutations is high, thus facilitating a switch to a novel, invading sex-determination system. The sex chromosome systems of therian mammals and carinate birds may have escaped this cycle, either because they have accumulated several sexually antagonistic alleles and/or accumulated a high proportion of haplo-sufficient genes on the Y (W) chromosome. Purifying selection might now prevent the Y (W) from losing haplo-insufficient genes [135,136]. In this case, the sex-determining supergene might remain more fit than any novel sex-determining haplotype, and thus might be difficult to replace.

### 3.1. Number of Genes in the Sex-Determination Network

Despite decades of research in humans and mice, the full complement of genes involved in mammalian sex-determination and differentiation is still unknown. The role of dozens of genes is now at least partially understood [137], but the genetic basis for most disorders of human sexual development remains unknown [138]. Furthermore, over time, the gene regulatory network for sex-determination is likely to evolve, and the set of genes capable of mutating to become the master sex-determination gene is likely to change. So, we might expect that a very large number of genes could be involved in vertebrate sex-determination, and that some genes controlling sex-determination in fish might be different from those in the mammalian gene regulatory network.

Some have argued that the number of genes capable of being the master sex-determination locus is limited because the sex chromosome systems characterized to date involve a small handful of genes [139]. In particular, DM domain genes have become the primary sex-determination mechanism in several independent lineages [140]. However, this conclusion may be the result of confirmation bias, because researchers typically search the regions of differentiation between males and females for candidate genes already known to be critically involved in the sex-determination network. Functional studies of these candidate genes frequently confirm their role in the sex-determination network, leading to a somewhat circular conclusion. We argue that our knowledge of the sex-determination network is still quite limited, which therefore constrains our ability to search for candidate genes within any given genomic interval. 

So far, the cichlid data suggest a great diversity of sex-determination mechanisms. There are only a few instances of convergence on the same genomic interval. ZW systems have evolved on LG3 in both tilapias and Lake Malawi haplochromines, but the mapped intervals are still large, and contain many genes. Both ZW and XY systems have evolved repeatedly on LG5 and on LG7, but the causative variation has not been identified. LG20 seems to be influential in contributing to sex-determination in both Oreochromini and Lake Malawi haplochromines. XY systems have evolved on LG23 in both Oreochromini and Lake Victoria haplochromines, but it is not known if both are due to mutations in the *anti-Müllerian hormone* gene. Many other sex-determination regions map to different chromosomes, *prima facie* evidence that they involve different genes. Furthermore, careful examination of many of these regions has failed to identify any of the usual candidate genes. This suggests that the sex-determination network is either larger than expected, or uses a different complement of genes in fishes. 

### 3.2. The Landscape of Sexual Antagonism

Theory suggests that sexually antagonistic alleles drive the evolution of new sex chromosomes. If cichlids have an unusually high rate of sex chromosome turnover, it may be because they have high levels of sexually antagonistic selection. All cichlids show a high degree of parental care and many show high levels of sexual dimorphism. The ancestral state for the group is bi-parental substrate spawning, in which monogamous pairs cooperate to defend a territory and protect the young [141]. Mouth-brooding has evolved in several lineages, including the hyper-diverse haplochromines in East Africa [141]. In the most derived forms of mouth-brooding, females pick up their eggs immediately after spawning, and incubate the eggs/larvae for up to three weeks, without any help from the male. This high degree of parental care is correlated with high levels of sexually antagonistic selection, which manifests as differences in behavior, size, and pigmentation between males and females. The genetic basis for sexual antagonism is likely to involve differences in the expression of thousands of genes scattered across the genome [142]. Sex differences in gene expression are common in many organisms and, depending on the tissue, organ, and time point selected, can represent more than half of the genome [143,144,145]. The most detailed study estimated that 8% of *Drosophila melanogaster* genes met the criteria for sexual antagonism [146]. If cichlids have at least a similar proportion of sexually antagonistic genes, there could be ~2 genes segregating sexual antagonistic variation in every megabase across the cichlid genome. 

The landscape of sexually antagonistic selection is constantly changing. Most loci are fixed for an allele that is a compromise between the optimal expression for males and females. New mutations that disrupt this compromise will be removed by stabilizing selection. A few loci segregate alleles with different levels of expression that might remain polymorphic through a combination of balancing selection and drift. While we currently do not know how many sexually antagonistic loci are polymorphic at any given time, it seems reasonable to believe that the landscape of sexually antagonistic selection might depend on the degree of sexual differentiation, the history of sexual antagonism, and the molecular mechanisms that have been selected to differentiate gene expression and reduce sexually antagonistic selection. 

### 3.3. Predicting the Appearance of New Sex Chromosomes

If we knew the landscape of sexually antagonistic selection across the genome and the location of genes that are part of the sex-determination regulatory network, we might be able to predict where new sex chromosomes might arise. Each of the nodes in the sex-determination network has a probability of becoming the next master sex-determination gene, but those that are closely linked to strong sexually antagonistic selection will become fixed most rapidly. Thus, each chromosome segment has a finite probability of becoming contained within the next sex chromosome.

**Figure 3:**
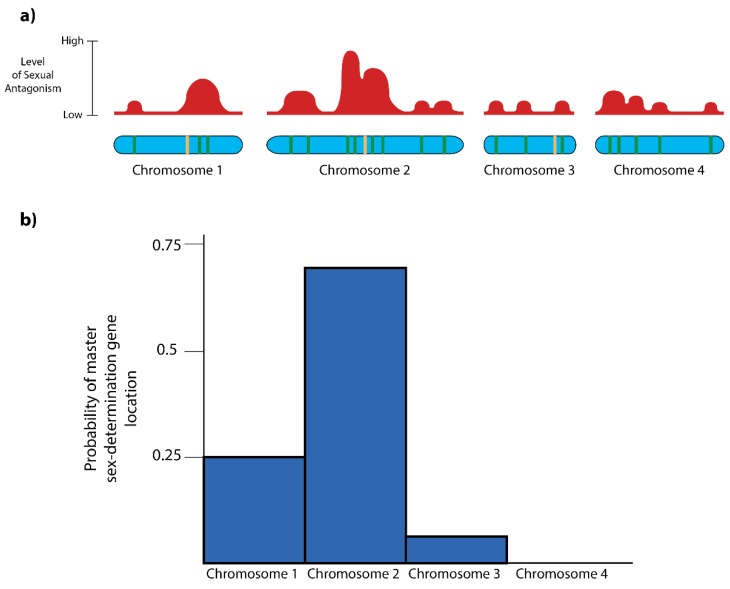
(**a**) Hypothetical genome with green bars on the chromosomes representing sexually antagonistic loci and tan bars representing nodes in the gene regulatory network for sex-determination. The density of sexual antagonistic alleles determines the level of sexual antagonism in the genome. (**b**) Probability function reflecting the likelihood that a particular chromosome becomes the next sex chromosome in a particular lineage.

Figure 3a depicts a hypothetical genome superimposing the polymorphic, sexually antagonistic landscape onto the sex-determination network. Even if we make the conservative assumption that the sexually antagonistic loci are distributed following a Poisson distribution and are not clustered due to selective forces, then, by chance, some regions—such as the right end of chromosome 1 and the middle of chromosome 2—have islands of polymorphic, sexually antagonistic alleles. Furthermore, some of these regions contain nodes in the sex-determination network. 

We might then make predictions about the evolution of sex-determination and sex chromosomes within these regions (Figure 3b). The probability that a particular locus becomes fixed as the master sex-determination system is a function of the combined strength of the polymorphic, sexually antagonistic alleles in the region; the recombination distance between those sexually antagonistic alleles and the sex-determination locus; the probability of a mutation creating a sex-determination allele at that node; and the probability of mutations occurring which convert monomorphic sexually antagonistic loci into polymorphic sexually antagonistic loci which can alleviate sexual conflict when linked to a sex-determination mechanism.

Importantly, this model predicts that many chromosome segments could be recruited to become sex chromosomes. The shape of the probability function in Figure 3b for a given group of organisms determines how often a chromosome could potentially be recruited as a sex chromosome. Furthermore, this probability function is dynamic over temporal and spatial scales as selection pressures change, so that different chromosome segments are recruited to become sex chromosomes in different lineages. 

We might expect that selection would favor the recruitment of sex-determination genes from regions of the genome with low recombination, because it would increase the association between sexually antagonistic variants and the new sex-determination allele. In fact, what matters is whether the average of the linked sexually antagonistic effects is favorable for the new sex-determination allele. Aside from LG3, which cytogenetic studies suggest has a low recombination rate [72,73,74,75,84], the cichlid sex-determination genes mapped to date do not fall in regions of the genome with ancestrally low levels of recombination. 

Most of the cichlid sex-determination systems identified so far appear to be associated with a putative inversion that created a single evolutionary stratum on a given chromosome. This suggests an important, perhaps critical, role for inversion mutations in the establishment of new sex chromosomes. As yet, we know very little about the patterns and rates of such mutations in the genome. It is important to note that no breakpoints for the putative inversions have been currently identified in cichlids and their presence is only inferred from the sharp boundaries between the highly differentiated regions and the rest of the genome.

## 4. Sex Chromosomes and Cichlid Speciation

It seems unlikely that any one mechanism will fully explain the rapid adaptive radiation of cichlids in East Africa. A variety of mechanisms are almost certainly contributing, including habitat adaptation, behavioral adaptation, and trophic adaptation [114]. However, the rapid evolution of sex chromosomes is likely to be an important additional factor in the speciation of cichlids. Rapid turnover of sex-determination genes alters the molecular interactions among genes in the sex-determination network. Incompatibilities arising from epistatic interactions within this network may contribute to reduced fitness of hybrids via gonadal dysgenesis, infertility, and inviability. Even in the absence of direct effects on hybrid fitness, the new genomic compartments created by these novel sex chromosomes may accelerate the divergence of species by increasing the opportunities for genetic conflicts [147].

## 5. Conclusions

Cichlids provide an excellent model system for understanding the evolution of young sex chromosomes as they present a diverse array of sex chromosomes at various stages of differentiation. The large number of sex chromosome systems among a closely related set of taxa provides a rich set of data points for testing theoretical models concerning the emergence, establishment, and evolution of novel sex chromosomes. It will be very interesting to see how many additional sex chromosome systems remain to be discovered in East African cichlids in particular, and in the family Cichlidae generally. 

In conclusion, we hypothesize that the rich diversity of cichlid sex chromosomes has arisen because of the widespread distribution of sexually antagonistic loci across the genome. There are frequent opportunities for the development of a new sex chromosome, because there is usually at least one sexually antagonistic locus near each of the dozens of genes that could potentially be co-opted to become a novel sex-determination locus. Frequent transitions among these sex-determination systems likely contribute to the rapid and fantastic diversity of sex chromosomes and species in cichlid fishes. 

## References

[1] Lindholm A., Breden F. (2002). Sex chromosomes and sexual selection in Poeciliid fishes. Am. Nat..

[2] Roberts R.B., Ser J.R., Kocher T.D. (2009). Sexual conflict resolved by invasion of a novel sex determiner in Lake Malawi cichlid fishes. Science.

[3] Charlesworth B., Coyne J.A., Barton N.H. (1987). The relative rates of evolution of sex chromosomes and autosomes. Am. Nat..

[4] Presgraves D.C. (2008). Sex chromosomes and speciation in *Drosophila*. Trends Genet..

[5] Haldane J.B.S. (1922). Sex ratio and unisexual sterility in hybrid animals. J. Genet..

[6] Bachtrog D. (2013). Y-chromosome evolution: Emerging insights into processes of Y-chromosome degeneration. Nat. Rev. Genet..

[7] Abbott J.K., Nordén A.K., Hansson B. (2017). Sex chromosome evolution: Historical insights and future perspectives. Proc. R. Soc. B Biol. Sci..

[8] Rice W.R. (1987). The accumulation of sexually antagonistic genes as a selective agent promoting the evolution of reduced recombination between primitive sex chromosomes. Evolution.

[9] Fisher R.A. (1931). The evolution of dominance. Biol. Rev..

[10] van Doorn G.S., Kirkpatrick M. (2010). Transitions between male and female heterogamety caused by sex-antagonistic selection. Genetics.

[11] Wright A.E., Darolti I., Bloch N.I., Oostra V., Sandkam B., Buechel S.D., Kolm N., Breden F., Vicoso B., Mank J.E. (2017). Convergent recombination suppression suggests role of sexual selection in guppy sex chromosome formation. Nat. Commun..

[12] Muller H. (1964). The relation of recombination to mutational advance. Mutat. Res..

[13] Bachtrog D., Kirkpatrick M., Mank J.E., McDaniel S.F., Pires J.C., Rice W., Valenzuela N. (2011). Are all sex chromosomes created equal?. Trends Genet..

[14] Koopman P., Gubbay J., Vivian N., Goodfellow P., Lovell-Badge R. (1991). Male development of chromosomally female mice transgenic for *Sry*. Nature.

[15] Cortez D., Marin R., Toledo-Flores D., Froidevaux L., Liechti A., Waters P.D., Grützner F., Kaessmann H. (2014). Origins and functional evolution of Y chromosomes across mammals. Nature.

[16] Honda T., Suzuki H., Itoh M. (1977). An unusual sex chromosome constitution found in the Amami spinous country-rat, *Tokudaia osimensis osimensis*. Jpn. J. Genet..

[17] Honda T., Suzuki H., Itoh M., Hayashi K. (1978). Karyotypical differences of the Amami spinous country-rats, *Tokudaia osimensis osimensis* obtained from two neighboring islands. Jpn. J. Genet..

[18] Sutou S., Mitsui Y., Tsuchiya K. (2001). Sex determination without the Y chromosome in two Japanese rodents *Tokudaia osimensis osimensis* and *Tokudaia osimensis* spp.. Mamm. Genome.

[19] Vorontsov N.N., Lyapunova E.A., Borissov Y.M., Dovgal V.E. (1980). Variability of sex chromosomes in mammals. Genetica.

[20] Kolomiets O.L., Vorontsov N.N., Lyapunova E.A., Mazurova T.F. (1991). Ultrastructure, meiotic behavior, and evolution of sex chromosomes of the genus *Ellobius*. Genetica.

[21] Just W., Baumstark A., Süß A., Graphodatsky A., Rens W., Schäfer N., Bakloushinskaya I., Hameister H., Vogel W. (2007). *Ellobius lutescens*: Sex determination and sex chromosome. Sex. Dev..

[22] Graves J.A.M. (2014). Avian sex, sex chromosomes, and dosage compensation in the age of genomics. Chromosom. Res..

[23] Stiglec R., Ezaz T., Graves J.A.M. (2007). A new look at the evolution of avian sex chromosomes. Cytogenet. Genome Res..

[24] Ellegren H. (2000). Evolution of the avian sex chromosomes and their role in sex determination. Trends Ecol. Evol..

[25] Ogawa A., Murata K., Mizuno S. (1998). The location of Z- and W-linked marker genes and sequence on the homomorphic sex chromosomes of the ostrich and the emu. Proc. Natl. Acad. Sci. USA.

[26] Gamble T. (2010). A review of sex determining mechanisms in geckos (Gekkota: Squamata). Sex. Dev..

[27] Bachtrog D., Mank J.E., Peichel C.L., Kirkpatrick M., Otto S.P., Ashman T.-L., Hahn M.W., Kitano J., Mayrose I., Ming R. (2014). The tree of sex consortium sex determination: Why so many ways of doing it?. PLoS Biol..

[28] Vicoso B., Bachtrog D. (2015). Numerous transitions of sex chromosomes in diptera. PLoS Biol..

[29] Devlin R.H., Nagahama Y. (2002). Sex determination and sex differentiation in fish: An overview of genetic, physiological, and environmental influences. Aquaculture.

[30] Gomelsky B. (2003). Chromosome set manipulation and sex control in common carp: A review. Aquat. Living Resour..

[31] Collares-Pereira M.J., Próspero M.I., Biléu R.I., Rodrigues E.M. (1998). *Leuciscus* (Pisces, Cyprinidae) karyotypes: Transect of Portuguese populations. Genet. Mol. Biol..

[32] Volff J.-N., Schartl M. (2001). Variability of genetic sex determination in Poeciliid fishes. Genetica.

[33] Artoni R.F., Venere P.C., Bertollo L.A.C. (1998). A heteromorphic ZZ/ZW sex chromosome system in fish, genus *Hypostomus* (Loricariidae). Cytologia.

[34] Andreata A.A., de Almeida-Toledo L.F., Oliveira C., de Almeida Toledo Filho S. (1992). Chromosome studies in Hypoptopomatinae (Pisces, Siluriformes, Loricariidade). Cytologia.

[35] Pennell M.W., Mank J.E., Peichel C.L. (2018). Transitions in sex determination and sex chromosomes across vertebrate species. Mol. Ecol..

[36] Uwa H., Ojima Y. (1981). Detailed and banding karyotype analyses the medaka, *Oryzias latipes* in cultured cells. Proc. Jpn. Acad. Ser. B.

[37] Uwa H., Iwanatsu T., Ojima Y. (1981). Karyotype and banding analyses of *Oryzias celebensis* (Oryziatidae, Pisces) in cultured cells. Proc. Jpn. Acad. Ser. B.

[38] Matsuda M., Nagahama Y., Shinomiya A., Sato T., Matsuda C., Kobayashi T., Morrey C.E., Shibata N., Asakawa S., Shimizu N. (2002). *DMY* is a Y-specific DM-domain gene required for male development in the medaka fish. Nature.

[39] Nanda I., Kondo M., Hornung U., Asakawa S., Winkler C., Shimizu A., Shan Z., Haaf T., Shimizu N., Shima A. (2002). A duplicated copy of *DMRT1* in the sex-determining region of the Y chromosome of the medaka, *Oryzias latipes*. Proc. Natl. Acad. Sci. USA.

[40] Nagai T., Takehana Y., Hamaguchi S., Sakaizumi M. (2008). Identification of the sex-determining locus in the Thai medaka, *Oryzias minutillus*. Cytogenet. Genome Res..

[41] Takehana Y., Naruse K., Hamaguchi S., Sakaizumi M. (2007). Evolution of ZZ/ZW and XX/XY sex-determination systems in the closely related medaka species, *Oryzias hubbsi* and *O. dancena*. Chromosoma.

[42] Takehana Y., Hamaguchi S., Sakaizumi M. (2008). Different origins of ZZ/ZW sex chromosomes in closely related medaka fishes, *Oryzias javanicus* and *O. hubbsi*. Chromosom. Res..

[43] Takehana Y., Matsuda M., Myosho T., Suster M.L., Kawakami K., Shin-I T., Kohara Y., Kuroki Y., Toyoda A., Fujiyama A. (2014). Co-option of *Sox3* as the male-determining factor on the Y chromosome in the fish *Oryzias dancena*. Nat. Commun..

[44] Takehana Y., Naruse K., Sakaizumi M. (2005). Molecular phylogeny of the medaka fishes genus *Oryzias* (Beloniformes: Adrianichthyidae) based on nuclear and mitochondrial DNA sequences. Mol. Phylogenet. Evol..

[45] Setiamarga D.H.E., Miya M., Yamanoue Y., Azuma Y., Inoue J.G., Ishiguro N.B., Mabuchi K., Nishida M. (2009). Divergence time of the two regional medaka populations in Japan as a new time scale for comparative genomics of vertebrates. Biol. Lett..

[46] Kirchmaier S., Naruse K., Wittbrodt J., Loosli F. (2015). The genomic and genetic toolbox of the teleost medaka (*Oryzias latipes*). Genetics.

[47] Myosho T., Otake H., Masuyama H., Matsuda M., Kuroki Y., Fujiyama A., Naruse K., Hamaguchi S., Sakaizumi M. (2012). Tracing the emergence of a novel sex-determining gene in medaka, *Oryzias luzonensis*. Genetics.

[48] Hattori R.S., Murai Y., Oura M., Masuda S., Majhi S.K., Sakamoto T., Fernandino J.I., Somoza G.M., Yokota M., Strüssmann C. (2012). A Y-linked anti-Müllerian hormone duplication takes over a critical role in sex determination. Proc. Natl. Acad. Sci. USA.

[49] Kamiya T., Kai W., Tasumi S., Oka A., Matsunaga T., Mizuno N., Fujita M., Suetake H., Suzuki S., Hosoya S. (2012). A trans-species missense SNP in *Amhr2* is associated with sex determination in the tiger pufferfish, *Takifugu rubripes* (fugu). PLoS Genet..

[50] Reichwald K., Petzold A., Koch P., Downie B.R., Hartmann N., Pietsch S., Baumgart M., Chalopin D., Felder M., Bens M. (2015). Insights into sex chromosome evolution and aging from the genome of a short-lived fish. Cell.

[51] Yano A., Guyomard R., Nicol B., Jouanno E., Quillet E., Klopp C., Cabau C., Bouchez O., Fostier A., Guiguen Y. (2012). An immune-related gene evolved into the master sex-determining gene in rainbow trout, *Oncorhynchus mykiss*. Curr. Biol..

[52] Faber-Hammond J.J., Phillips R.B., Brown K.H. (2015). Comparative analysis of the shared sex-determination region (sdr) among salmonid fishes. Genome Biol. Evol..

[53] Meyer B.S., Matschiner M., Salzburger W. (2015). A tribal level phylogeny of Lake Tanganyika cichlid fishes based on a genomic multi-marker approach. Mol. Phylogenet. Evol..

[54] Dunz A.R., Schliewen U.K. (2013). Molecular phylogeny and revised classification of the haplotilapiine cichlid fishes formerly referred to as “Tilapia”. Mol. Phylogenet. Evol..

[55] Nagl S., Tichy H., Mayer W.E., Samonte I.E., McAndrew B.J., Klein J. (2001). Classification and phylogenetic relationships of African tilapiine fishes inferred from mitochondrial DNA sequences. Mol. Phylogenet. Evol..

[56] Klett V., Meyer A. (2002). What, if anything, is a *Tilapia*?—mitochondrial ND2 phylogeny of tilapiines and the evolution of parental care systems in the African cichlid fishes. Mol. Biol. Evol..

[57] Farias I.P., Ortí G., Sampaio I., Schneider H., Meyer A. (1999). Mitochondrial DNA phylogeny of the family Cichlidae: Monophyly and fast molecular evolution of the neotropical assemblage. J. Mol. Evol..

[58] Sparks J.S., Smith W.L. (2004). Phylogeny and biogeography of cichlid fishes (Teleostei: Perciformes: Cichlidae). Cladistics.

[59] McMahan C.D., Chakrabarty P., Sparks J.S., Smith W.M.L., Davis M.P. (2013). Temporal patterns of diversification across global cichlid biodiversity (Acanthomorpha: Cichlidae). PLoS ONE.

[60] Friedman M., Keck B.P., Dornburg A., Eytan R.I., Martin C.H., Darrin C., Wainwright P.C., Near T.J., Hulsey C.D. (2013). Molecular and fossil evidence place the origin of cichlid fishes long after Gondwanan rifting. Proc. Biol. Sci..

[61] Matschiner M., Musilová Z., Barth J.M.I., Starostová Z., Salzburger W., Steel M., Bouckaert R. (2017). Bayesian phylogenetic estimation of clade ages supports trans-Atlantic dispersal of cichlid fishes. Syst. Biol..

[62] Irisarri I., Singh P., Koblmüller S., Torres-Dowdall J., Henning F., Franchini P., Fischer C., Lemmon A.R., Lemmon E.M., Thallinger G.G. (2018). Phylogenomics uncovers early hybridization and adaptive loci shaping the radiation of Lake Tanganyika cichlid fishes. Nat. Commun..

[63] Schwarzer J., Misof B., Tautz D., Schliewen U.K. (2009). The root of the East African cichlid radiations. BMC Evol. Biol..

[64] Lee B.-Y., Penman D.J., Kocher T.D. (2003). Identification of a sex-determining region in Nile tilapia (*Oreochromis niloticus*) using bulked segregant analysis. Anim. Genet..

[65] Ezaz M.T., Harvey S.C., Boonphakdee C., Teale A.J., McAndrew B.J., Penman D.J. (2004). Isolation and physical mapping of sex-linked AFLP markers in nile tilapia (*Oreochromis niloticus* L.). Mar. Biotechnol..

[66] Lee B.-Y., Coutanceau J.-P., Ozouf-Costaz C., D’Cotta H., Baroiller J.-F., Kocher T.D. (2011). Genetic and physical mapping of sex-linked AFLP markers in Nile tilapia (*Oreochromis niloticus*). Mar. Biotechnol..

[67] Cnaani A., Lee B.-Y., Zilberman N., Ozouf-Costaz C., Hulata G., Ron M., D’Hont A., Baroiller J.-F., D’Cotta H., Penman D.J. (2008). Genetics of sex determination in tilapiine species. Sex. Dev..

[68] Palaiokostas C., Bekaert M., Khan M.G.Q., Taggart J.B., Gharbi K., McAndrew B.J., Penman D.J. (2013). Mapping and validation of the major sex-determining region in Nile tilapia (*Oreochromis niloticus* L.) using RAD sequencing. PLoS ONE.

[69] Palaiokostas C., Bekaert M., Khan M.G.Q., Taggart J.B., Gharbi K., McAndrew B.J., Penman D.J. (2015). A novel sex-determining QTL in Nile tilapia (*Oreochromis niloticus*). BMC Genomics.

[70] Gammerdinger W.J., Conte M.A., Acquah E.A., Roberts R.B., Kocher T.D. (2014). Structure and decay of a proto-Y region in tilapia, *Oreochromis niloticus*. BMC Genomics.

[71] Gammerdinger W.J., Conte M.A., Baroiller J.-F., D’Cotta H., Kocher T.D. (2016). Comparative analysis of a sex chromosome from the blackchin tilapia, *Sarotherodon melanotheron*. BMC Genomics.

[72] Ocalewicz K., Mota-Velasco J.C., Campos-Ramos R., Penman D.J. (2009). FISH and DAPI staining of the synaptonemal complex of the Nile tilapia (*Oreochromis niloticus*) allow orientation of the unpaired region of bivalent 1 observed during early pachytene. Chromosom. Res..

[73] Campos-Ramos R., Harvey S.C., Penman D.J. (2009). Sex-specific differences in the synaptonemal complex in the genus *Oreochromis* (Cichlidae). Genetica.

[74] Foresti F., Oliveira C., Galetti Junior P.M., Almeida-Toledo L.F.D. (1993). Synaptonemal complex analysis in spermatocytes of tilapia, *Oreochromis niloticus* (Pisces, Cichlidae). Genome.

[75] Carrasco L.A.P., Penman D.J., Bromage N. (1999). Evidence for the presence of sex chromosomes in the Nile tilapia (*Oreochromis niloticus*) from synaptonemal complex analysis of XX, XY and YY genotypes. Aquaculture.

[76] Conte M.A., Gammerdinger W.J., Bartie K.L., Penman D.J., Kocher T.D. (2017). A high quality assembly of the Nile tilapia (*Oreochromis niloticus*) genome reveals the structure of two sex determination regions. BMC Genomics.

[77] Harvey S.C., Powell S.F., Kennedy D.D., Mcandrew B.J., Penman D.J. (2002). Karyotype analysis of *Oreochromis mortimeri* (Trewavas) and *Sarotherodon melanotheron* (Rüppell). Aquac. Res..

[78] Majumdar K.C., McAndrew B.J. (1984). Relative DNA content of somatic nuclei and chromosomal studies in three genera, *Tilapia*, *Sarotherodon*, and *Oreochromis* of the tribe Tilapiini (Pisces, Cichlidae). Genetica.

[79] Cnaani A., Zilberman N., Tinman S., Hulata G., Ron M. (2004). Genome-scan analysis for quantitative trait loci in an F_2_ tilapia hybrid. Mol. Genet. Genomics.

[80] Lee B.-Y., Hulata G., Kocher T.D. (2004). Two unlinked loci controlling the sex of blue tilapia (*Oreochromis aureus*). Heredity.

[81] Thompson K.W. (1981). Karyotypes of six species of African Cichlidae (Pisces: Perciformes). Experientia.

[82] Campos-Ramos R., Harvey S.C., Masabanda J.S., Carrasco L.A.P., Griffin D.K., McAndrew B.J., Bromage N.R., Penman D.J. (2001). Identification of putative sex chromosomes in the blue tilapia, *Oreochromis aureus*, through synaptonemal complex and FISH analysis. Genetica.

[83] Cnaani A., Kocher T.D. (2008). Sex-linked markers and microsatellite locus duplication in the cichlid species *Oreochromis tanganicae*. Biol. Lett..

[84] Harvey S.C., Campos-Ramos R., Kennedy D.D., Ezaz M.T., Bromage N.R., Griffin D.K., Penman D.J. (2002). Karyotype evolution in Tilapia: Mitotic and meiotic chromosome analysis of *Oreochromis karongae* and *O. niloticus* x *O. karongae* hybrids. Genetica.

[85] Mota-Velasco J.C., Ferreira I.A., Cioffi M.B., Ocalewicz K., Campos-Ramos R., Shirak A., Lee B.-Y., Martins C., Penman D.J. (2010). Characterisation of the chromosome fusions in *Oreochromis karongae*. Chromosom. Res..

[86] Shirak A., Palti Y., Cnaani A., Korol A., Hulata G., Ron M., Avtalion R.R. (2002). Association between loci with deleterious alleles and distorted sex ratios in an inbred line of tilapia (*Oreochromis aureus*). J. Hered..

[87] Cnaani A., Hallerman E.M., Ron M., Weller J.I., Indelman M., Kashi Y., Gall G.A.E., Hulata G. (2003). Detection of a chromosomal region with two quantitative trait loci, affecting cold tolerance and fish size, in an F_2_ tilapia hybrid. Aquaculture.

[88] Eshel O., Shirak A., Weller J.I., Slossman T., Hulata G., Cnaani A., Ron M. (2011). Fine-mapping of a locus on linkage group 23 for sex determination in Nile tilapia (*Oreochromis niloticus*). Anim. Genet..

[89] Eshel O., Shirak A., Weller J.I., Hulata G., Ron M. (2012). Linkage and physical mapping of sex region on LG23 of Nile tilapia (*Oreochromis niloticus*). G3.

[90] Eshel O., Shirak A., Dor L., Band M., Zak T., Markovich-Gordon M., Chalifa-Caspi V., Feldmesser E., Weller J.I., Seroussi E. (2014). Identification of male-specific amh duplication, sexually differentially expressed genes and microRNAs at early embryonic development of Nile tilapia (*Oreochromis niloticus*). BMC Genomics.

[91] Li M., Sun Y., Zhao J., Shi H., Zeng S., Ye K., Jiang D., Zhou L., Sun L., Tao W. (2015). A tandem duplicate of anti-müllerian hormone with a missense SNP on the Y Chromosome is essential for male sex determination in Nile Tilapia, *Oreochromis niloticus*. PLoS Genet..

[92] Gammerdinger W.J., Conte M.A., Sandkam B.A., Penman D.J., Kocher T.D. Characterization of sex chromosomes in three deeply diverged species of Pseudocrenilabrinae (Teleostei: Cichlidae). Hydrobiologia.

[93] Kide N.G., Dunz A., Agnèse J.F., Dilyte J., Pariselle A., Carneiro C., Correia E., Brito J.C., Yarba L.O., Kone Y., Durand J.-D. (2016). Cichlids of the Banc d’Arguin National Park, Mauritania: Insight into the diversity of the genus *Coptodon*. J. Fish Biol..

[94] Sofy H.I., Layla A.M., Iman M.K.A. (2008). Karyotypic diversity of some tilapia species. Nat. Sci..

[95] Ferreira I.A., Poletto A.B., Kocher T.D., Mota-Velasco J.C., Penman D.J., Martins C. (2010). Chromosome evolution in african cichlid fish: Contributions from the physical mapping of repeated DNAs. Cytogenet. Genome Res..

[96] Swanepoel A. (1990). ’n Vergelykende studie van die kariotipes van *Tilapia rendalli*, *Tilapia sparrmanii* en *Oreochromis mossambicus* (Cichlidae). Master’s Thesis.

[97] Michele J.L., Takahashi C.S. (1977). Comparative cytology of *Tilapia rendalli* and *Geophagus brasiliensis* (Cichlidae, Pisces). Cytologia.

[98] Vervoort A. (1980). The karyotypes of seven species of *Tilapia* (Teleostei: Cichlidae). Cytologia.

[99] Ferreira I.A., Martins C. (2008). Physical chromosome mapping of repetitive DNA sequences in Nile tilapia *Oreochromis niloticus*: Evidences for a differential distribution of repetitive elements in the sex chromosomes. Micron.

[100] Rizzon C., Marais G., Gouy M., Biémont C. (2002). Recombination rate and the distribution of transposable elements in the *Drosophila melanogaster* genome. Genome Res..

[101] Jensen-Seaman M., Furey T.S., Payseur B.A., Lu Y., Roskin K.M., Chen C.-F., Thomas M.A., Haussler D., Jacob H.J. (2004). Comparative recombination rates in the rat, mouse, and human genomes. Genome Res..

[102] Charlesworth B., Sniegowski P., Stephan W. (1994). The evolutionary dynamics of repetitive DNA in eukaryotes. Nature.

[103] Cohen A.S., Soreghan M.J., Scholz C.A. (1993). Estimating the age of formation of lakes: An example from Lake Tanganyika, East African Rift system. Geology.

[104] Turner G.F., Seehausen O., Knight M.E., Allender C.J., Robinson R.L. (2001). How many species of cichlid fishes are there in African lakes?. Mol. Ecol..

[105] Takahashi K., Terai Y., Nishida M., Okada N. (2001). Phylogenetic relationships and ancient incomplete lineage sorting among cichlid fishes in Lake Tanganyika as revealed by analysis of the insertion of retroposons. Mol. Biol. Evol..

[106] Gammerdinger W.J., Conte M.A., Sandkam B.A., Ziegelbecker A., Koblmüller S., Kocher T.D. (2018). Novel sex chromosomes in three cichlid fishes from Lake Tanganyika. J. Hered..

[107] Salzburger W., Meyer A., Baric S., Verheyen E., Sturmbauer C. (2002). Phylogeny of the Lake Tanganyika cichlid species flock and its relationship to the Central and East African haplochromine cichlid fish faunas. Syst. Biol..

[108] Koblmüller S., Duftner N., Katongo C., Phiri H., Sturmbauer C. (2005). Ancient divergence in bathypelagic Lake Tanganyika deepwater cichlids: Mitochondrial phylogeny of the tribe Bathybatini. J. Mol. Evol..

[109] Takahashi T., Sota T. (2016). A robust phylogeny among major lineages of the East African cichlids. Mol. Phylogenet. Evol..

[110] Ozouf-Costaz C., Coutanceau J.-P., Bonillo C., Mercot H., Fermon Y., Guidi-Rontani C. (2017). New insights into the chromosomal differentiation patterns among cichlids from Africa and Madagascar. Cybium Int. J. Ichthyol..

[111] Koblmüller S., Schliewen U.K., Duftner N., Sefc K.M., Katongo C., Sturmbauer C. (2008). Age and spread of the haplochromine cichlid fishes in Africa. Mol. Phylogenet. Evol..

[112] Genner M.J., Seehausen O., Lunt D.H., Joyce D.A., Shaw P.W., Carvalho G.R., Turner G.F. (2007). Age of cichlids: New dates for ancient lake fish radiations. Mol. Biol. Evol..

[113] Salzburger W., Mack T., Verheyen E., Meyer A. (2005). Out of Tanganyika: Genesis, explosive speciation, key-innovations and phylogeography of the haplochromine cichlid fishes. BMC Evol. Biol..

[114] Kocher T.D. (2004). Adaptive evolution and explosive speciation: The cichlid fish model. Nat. Rev. Genet..

[115] Böhne A., Wilson C.A., Postlethwait J.H., Salzburger W. (2016). Variations on a theme: Genomics of sex determination in the cichlid fish *Astatotilapia burtoni*. BMC Genomics.

[116] Roberts N.B., Juntti S.A., Coyle K.P., Dumont B.L., Stanley M.K., Ryan A.Q., Fernald R.D., Roberts R.B. (2016). Polygenic sex determination in the cichlid fish, *Astatotilapia burtoni*. BMC Genomics.

[117] Sturmbauer C., Baric S., Salzburger W., Ruber L., Verheyen E. (2001). Lake level fluctuations synchronized genetic divergence of cichlid fishes in African lakes. Mol. Biol. Evol..

[118] Kocher T.D., Conroy J.A., McKaye K.R., Stauffer J.R., Lockwood S.F. (1995). Evolution of NADH Dehydrogenase Subunit 2 in East African cichlid fish. Mol. Phylogenet. Evol..

[119] Meyer A., Kocher T.D., Basasibwaki P., Wilson A.C. (1990). Monophyletic orign of Lake Victoria cichlid fishes suggested by mitochondrial DNA sequences. Nature.

[120] Streelman J.T., Albertson R.C., Kocher T.D. (2003). Genome mapping of the orange blotch colour pattern in cichlid fishes. Mol. Ecol..

[121] Ser J.R., Roberts R.B., Kocher T.D. (2010). Multiple interacting loci control sex determination in Lake Malawi cichlid fish. Evolution.

[122] Parnell N.F., Streelman J.T. (2013). Genetic interactions controlling sex and color establish the potential for sexual conflict in Lake Malawi cichlid fishes. Heredity.

[123] O’Quin C.T. (2014). The Genetic Basis of Pigment Pattern Differentiation in Lake Malawi African Cichlids. Ph.D. Thesis.

[124] Peterson E.N., Cline M.E., Moore E.C., Roberts N.B., Roberts R.B. (2017). Genetic sex determination in *Astatotilapia calliptera*, a prototype species for the Lake Malawi cichlid radiation. Sci. Nat..

[125] Clark F.E., Conte M.A., Ferreira-Bravo I.A., Poletto A.B., Martins C., Kocher T.D. (2017). Dynamic sequence evolution of a sex-associated B chromosome in Lake Malawi cichlid fish. J. Hered..

[126] Martis M.M., Klemme S., Banaei-Moghaddam A.M., Blattner F.R., Macas J., Schmutzer T., Scholz U., Gundlach H., Wicker T., Simkova H. (2012). Selfish supernumerary chromosome reveals its origin as a mosaic of host genome and organellar sequences. Proc. Natl. Acad. Sci. USA.

[127] Valente G.T., Conte M.A., Fantinatti B.E.A., Cabral-De-Mello D.C., Carvalho R.F., Vicari M.R., Kocher T.D., Martins C. (2014). Origin and evolution of B chromosomes in the cichlid fish *Astatotilapia latifasciata* based on integrated genomic analyses. Mol. Biol. Evol..

[128] Verheyen E., Salzburger W., Snoeks J., Meyer A. (2003). Origin of the superflock of cichlid fishes from Lake Victoria, East Africa. Science.

[129] Kudo Y., Nikaido M., Kondo A., Suzuki H., Yoshida K., Kikuchi K., Okada N. (2015). A microsatellite-based genetic linkage map and putative sex-determining genomic regions in Lake Victoria cichlids. Gene.

[130] Feulner P.G.D., Schwarzer J., Haesler M.P., Meier J.I., Seehausen O. (2018). A dense linkage map of Lake Victoria cichlids improved the *Pundamilia* genome assembly and revealed a major QTL for sex-determination. G3 Genes Genomes Genet..

[131] Yoshida K., Terai Y., Mizoiri S., Aibara M., Nishihara H., Watanabe M., Kuroiwa A., Hirai H., Hirai Y., Matsuda Y., Okada N. (2011). B chromosomes have a functional effect on female sex determination in Lake Victoria cichlid fishes. PLoS Genet..

[132] Blaser O., Neuenschwander S., Perrin N. (2014). Sex-chromosome turnovers: The hot-potato model. Am. Nat..

[133] Blaser O., Grossen C., Neuenschwander S., Perrin N. (2012). Sex-chromsome turnovers induced by deleterious mutation load. Evolution.

[134] Rice W.R. (1986). On the instability of polygenic sex determination: The effect of sex-specific selection. Evolution.

[135] Bellott D.W., Hughes J.F., Skaletsky H., Brown L.G., Pyntikova T., Cho T.-J., Koutseva N., Zaghlul S., Graves T., Rock S. (2014). Mammalian Y chromosomes retain widely expressed dosage-sensitive regulators. Nature.

[136] White M.A., Kitano J., Peichel C.L. (2015). Purifying selection maintains dosage-sensitive genes during degeneration of the threespine stickleback Y chromosome. Mol. Biol. Evol..

[137] Eggers S., Ohnesorg T., Sinclair A. (2014). Genetic regulation of mammalian gonad development. Nat. Rev. Endocrinol..

[138] Eggers S., Sinclair A. (2012). Mammalian sex determination-insights from humans and mice. Chromosom. Res..

[139] Graves J.A.M., Peichel C.L. (2010). Are homologies in vertebrate sex determination due to shared ancestry or to limited options?. Genome Biol..

[140] Matson C.K., Zarkower D. (2012). Sex and the singular DM domain: Insights into sexual regulation, evolution and plasticity. Nat. Rev. Genet..

[141] Goodwin N.B., Balshine-Earn S., Reynolds J.D. (1998). Evolutionary transitions in parental care in cichlid fish. Proc. Biol. Sci..

[142] Mank J.E. (2009). Sex chromosomes and the evolution of sexual dimorphism: Lessons from the genome. Am. Nat..

[143] Chen S., Zhang G., Shao C., Huang Q., Liu G., Zhang P., Song W., An N., Chalopin D., Volff J.-N. (2014). Whole-genome sequence of a flatfish provides insights into ZW sex chromosome evolution and adaptation to a benthic lifestyle. Nat. Genet..

[144] Ranz J., Castillo-Davis C., Meiklejohn C., Hartl D. (2003). Sex-dependent gene expression and evolution of the *Drosophila* transcriptome. Science.

[145] Ellegren H., Parsch J. (2007). The evolution of sex-biased genes and sex-biased gene expression. Nat. Rev. Genet..

[146] Innocenti P., Morrow E.H. (2010). The sexually antagonistic genes of *Drosophila melanogaster*. PLoS Biol..

[147] Crespi B., Nosil P. (2013). Conflictual speciation: Species formation via genomic conflict. Trends Ecol. Evol..

